# Overview of MicroRNA Expression in Predicting Response to Neoadjuvant Therapies in Human Epidermal Growth Receptor-2 Enriched Breast Cancer – A Systematic Review

**DOI:** 10.1177/11782234221086684

**Published:** 2022-03-22

**Authors:** Matthew G Davey, Martin S Davey, Vinitha Richard, William Wyns, Osama Soliman, Nicola Miller, Aoife J Lowery, Michael J Kerin

**Affiliations:** 1 Discipline of Surgery, Lambe Institute for Translational Research, National University of Ireland, Galway, Galway, Ireland; 2 Precision Cardio-Oncology Research Enterprise (P-CORE), National University of Ireland, Galway, Galway, Ireland; 3 Department of Surgery, Galway University Hospitals, Galway, Ireland; 4 Discipline of Cardiology, CORRIB Core Laboratory, National University of Ireland, Galway, Galway, Ireland

**Keywords:** Breast cancer, microRNA, non-coding RNA, pathological complete response, personalised medicine, precision medicine

## Abstract

**Purpose:**

Increased appreciation of the human epidermal growth factor receptor-2 (HER2/neu) signalling pathway has led to the development of targeted therapeutic agents used in conjunction with chemotherapy to improve outcomes for HER2 overexpressing (HER2+) breast cancer. For neoadjuvant therapy, response rates can be unpredictable – novel biomarkers predicting effectiveness are required to enhance oncological outcomes for these patients, and microRNA may prove effective. Our objective was to identify microRNA (miRNA) expression patterns predictive of response to neoadjuvant chemotherapy (NAC) and/or anti-HER2 targeted therapies in patients being treated for early-stage HER2+ breast cancer.

**Methods:**

A search was performed of the PUBMED, SCOPUS, Web of Science, and EMBASE in accordance to Preferred Reporting Items for Systematic Review and Meta-Analyses (PRISMA) guidelines.

**Results:**

Overall, 15 studies including 1335 patients were included. These studies highlighted an expression profile of 73 miRNA and their ability to predict tumour response to neoadjuvant therapies was correlated. Results from 11 studies were in relation to circulatory miRNA and 4 studies included data from tumour tissue. Overall, upregulation and downregulation of 41 miRNA and 29 miRNA, respectively, predicted differential response to neoadjuvant therapy. Expression levels of 3 miRNA (miR-21, miR-210, and miR-376c-3p) were inconclusive in predicting therapeutic response, while ‘aberrant’ expression of circulating miR-199a predicted pathological complete response (pCR) to NAC.

**Conclusions:**

This systematic review outlines expression patterns of a number of miRNA which correlate with response to NAC and/or anti-HER2 therapies. Future translational research evaluating predictive biomarkers of primary response to neoadjuvant therapy in HER2+ breast cancer may consider these results.

## Introduction

In the western world, 1 in 8 women will face a breast cancer diagnosis in their lifetime.^1^ Nevertheless, breast cancer remains a common cause of cancer-related mortality, despite favourable prognoses anticipated for the majority.^2^ The molecular era has revolutionised the management paradigm and enhanced clinical outcomes by increasing our understanding of the biological processes driving breast cancer development, proliferation, and metastases. Appraisal of genomic and biological properties highlight the importance of molecular signalling in tumour biology and in personalising therapeutic decision making,^3-6^ while also providing prognostic information on the basis of the molecular phenotype of cancers.^7^ Such advances have facilitated the clinical substratification of the disease into 4 biologically distinct, clinically relevant, molecular subtypes: Luminal A, Luminal B, Triple Negative, and Human Epidermal Growth Factor Receptor-2 (HER2/neu) overexpressing breast cancers.^8^

In recent times, our understanding of the HER2/neu signalling pathway has facilitated the development of targeted therapeutic agents. Monoclonal antibodies capable of targeting the HER2/neu receptor and oral tyrosine kinase inhibitor that reversely inhibits HER1, HER2, and epidermal growth factor receptor kinases are now used in conjunction with conventional chemotherapeutic drugs to improve clinical and oncological outcomes.^9,10^ The combined prescription of Trastuzumab, Carboplatin, and Docetaxel (or ‘combination TCH’) results in previously unprecedented and favourable survival outcomes within the patient cohort with tumours with HER2/neu enriched signalling.^11^ Interestingly, tumour sensitivity to neoadjuvant chemotherapy (NAC) is an important prognostic parameter in breast cancer,^12^ with biomarkers such as pathological complete response (pCR) (defined as the absence of residual disease or eradication of invasive disease following neoadjuvant therapy)^13,14^ being associated with positive predictive value in relation to survival outcomes, with those achieving pCR exhibiting improved long-term survival.^12,15^ While pCR rates of as high as 70% have been described in the setting of HER2/neu enriched disease,^16,17^ modern translational research efforts have focused on identifying novel reliable and sensitive biomarkers which may rival current standard clinicopathologic markers (ie, oestrogen [ER], progesterone [PgR], HER2/neu receptor status, Ki-67 expression profiles, multigene expression assays) in informing oncological outcomes,^5,6,15,18-20^ such as response to neoadjuvant therapies. Nevertheless, there remains a paucity of practical biomarkers capable of predicating treatment outcomes in HER2/neu overexpressing breast cancer.

Micro-ribonucleic acids (or miRNAs) are small, non-coding molecules which are responsible for the regulation of genetic and protein expression through influencing post-transcriptional cellular activity.^21-24^ MiRNAs play roles in several biological and cellular processes, such as apoptosis, cell-cycle control, proliferation, and differentiation,^25^ while also having a role in cancer development and progression: oncogenic miRNA (oncomirs) encourage tumorigenesis, while tumour suppressor miRNA target oncogenes in the post-transcriptional phase, disrupting cancer cell proliferation.^26^ Previous authors suggest these small non-coding biomarkers may be clinically pertinent in deciphering those likely to achieve pCR to NAC for locally advanced, primary breast cancer.^27-30^ Accordingly, the aim of the current systematic review was to identify miRNA expression patterns which may be useful in predicting response to NAC and/or anti-HER2 targeted therapies in patients treated with curative intent for early-stage HER2 overexpressing (HER2+) breast cancer. Second, we wished to review the previous studies, their methodology, and their results for assessing miRNA expression profiles in predicting response to neoadjuvant therapy in HER2+ breast cancer.

## Methods

### Literature search

A formal systematic search was performed of the PUBMED, SCOPUS, Web of Science, and EMBASE databases in accordance to the Preferred Reporting Items for Systematic Review and Meta-Analyses (PRISMA) checklist.^31^ An initial predefined search strategy was outlined by the authors at the initiation of the study and supervised by the senior authors (A.J.L. and M.J.K.). Two authors (M.G.D. and M.S.D.) conducted an independent and comprehensive search of the 4 aforementioned databases for studies suitable for inclusion in this systematic review, the latest of which occurred in December 2020. Discrepancies in opinion between authors were arbitrated by a third author (V.R.). The search terms (breast cancer), (HER2), (biomarker), and (response) were all linked with the Boolean operator ‘AND’. Subsequent outcomes were combined from the 4 databases and duplicate studies were removed. A second independent search using the search terms (microrna) OR (mirna) AND (HER2) OR (HER2/neu) OR (errbb2) AND (breast cancer) AND (neoadjuvant therapies) AND (response) were all linked with the Boolean operator ‘AND’. Manuscripts published in languages other than the English language were excluded. Studies were not restricted based on year of publication. Proceedings from academic conferences and their abstracts were included. All studies had their titles screened initially and studies considered to be relevant had their abstracts reviewed. Full texts of remaining studies were assessed for relevance. The Quality Assessment of Diagnostic Accuracy Studies-2 (Quadras-2) was used to determine the diagnostic quality of included miRNAs.^32^

### Predefined inclusion criteria

Studies meeting the following predefined inclusion criteria were considered for inclusion in this systematic review: (1) studies assessing miRNA expression as biomarkers of response to conventional NAC and/or targeted anti-HER2 therapies in the tumour tissue of patients with HER2+ breast cancer or (2) studies assessing the expression of miRNA in patient circulation pre, during, or after the time of NAC and/or targeted anti-HER2 therapies prescription within tumour or circulatory tissue in patients diagnosed with HER2+ breast cancer. Studies reporting on miRNA within several molecular subtypes including HER2+ cancers were included (data in relation to patients treated for HER2+ disease were extracted). Any study failing to meet these criteria was excluded.

### Predefined exclusion criteria

Studies meeting the following criteria were excluded from this analysis: (1) studies demonstrating the efficacy of miRNA as biomarkers of response to therapies in other breast cancer molecular subtypes only or other malignancies, (2) review articles, (3) editorials, (4) animal studies, or (5) studies reporting results in relation to the sensitivity of biomarkers other than miRNA. As described, 2 independent reviewers performed the search and assessed the retrieved studies for inclusion and exclusion criteria before extracting: (1) name of the first author, (2) year of publication, (3) tissue used in evaluation, (4) methodology and laboratory techniques, (5) miRNA appraised, and (6) degree of tumour response to NAC and/or targeted anti-HER2 treatment as described in the article. Studies reporting data from the same centre were evaluated for the duplication of patient data; studies determined to possess data overlapping with included studies were removed.

### Statistical analysis and definitions

Data were extracted from included studies. Clinicopathological data were presented as proportions using descriptive statistics. MiRNA expression levels and treatment characteristics were described using narrative statistics. Analysis was performed using Statistical Package for Social Sciences™ (SPSS™) version 26. A response to neoadjuvant therapies was defined as ‘an overall reduction in clinical, radiological, or pathological tumour size following neoadjuvant therapy’. As previously outlined, pCR was defined as the ‘absence of residual disease or eradication of invasive disease from the breast and/or axilla following neoadjuvant therapy’.^13,14^

## Results

### Literature search

**Figure 1.**
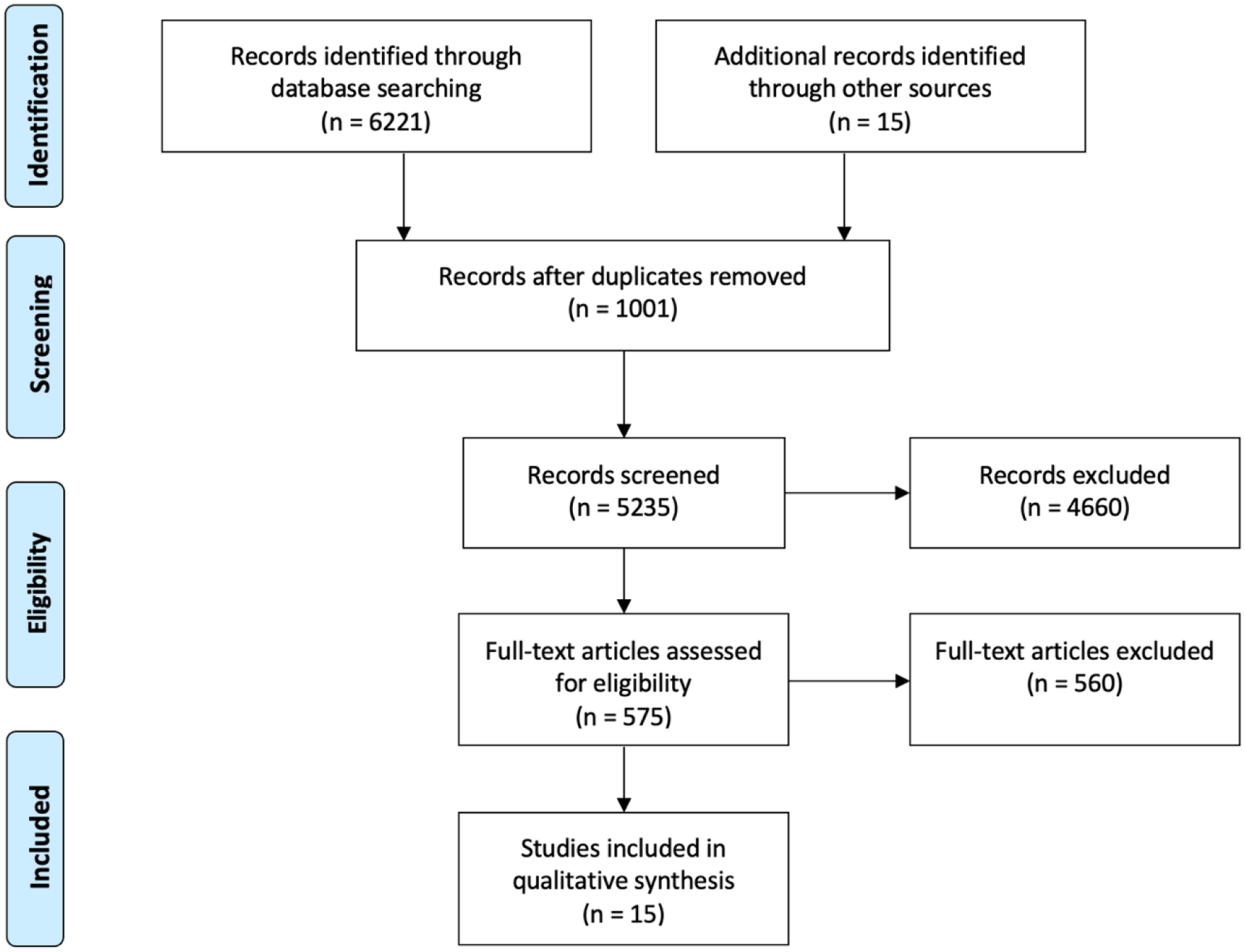
PRISMA flow diagram detailing the systematic search process.

The extensive literature search yielded a total of 6221 studies. Following the removal of 1001 duplicate studies, 5235 studies were screened for relevance for inclusion. Of these, 575 had their abstracts reviewed for relevance. Overall, 15 studies reporting on miRNA expression and their correlation to response to neoadjuvant therapies from in vivo human studies were included in this systematic review.^33-47^ A PRISMA flow diagram detailing the systematic search process is outlined in Figure 1.

### Included studies

There were 15 studies included in the current analysis, which included clinical samples from 1335 patients. Table 1 illustrates patient, translational research techniques and treatment characteristics for each of these studies. Of the 15 included studies, 11 reported results in relation to miRNA in circulation (ct-miRNA) (ie, blood, serum, or plasma) capable of predicting response to neoadjuvant therapies^33,34,36-40,42,43^ (Table 2). Four studies measured miRNA in tumour tissue and their association with response to neoadjuvant therapies (Table 3).^35,41,44,45^

**Table 1.** Included studies assessing miRNA expression and their role in predicting response to neoadjuvant therapies.

Author	Year	Country	Tissue	N	LOE	Neoadjuvant treatment	Timing of sampling	Technique	MiRNA selection
Di Cosimo et al^38^	2019	Italy	Plasma	429	Prospective (II); NeoALLTO trial (NCT: 00553358)	Trastuzumab, Lapatinib, and Paclitaxel	Initiation of NAC and after 2 weeks	qRT-PCR	miRNA array
Zhao et al^37^	2011	China	Plasma	27	Retrospective (III)	Epirubicin, Docetaxel, or Paclitaxel	Previously underwent NAC	qRT-PCR	Selected from literature
Di Cosimo et al^34^	2020	Italy	Plasma	429	Prospective (II); NeoALLTO trial (NCT: 00553358)	Trastuzumab, Lapatinib, and Paclitaxel	Initiation of NAC and after 2 weeks of NAC	qRT-PCR	miRNA array
Stevic et al^40^	2018	Germany	Plasma	211	Prospective (II); GeparSixto Trial (NCT: 01426880)	Docetaxel or Paclitaxel +/− Carboplatin	Pre-NAC and post-NAC	Western Blot/ELISA	TaqMan© miRNA array
Jung et al^43^	2012	US/Korea	Plasma	72	Prospective (II)	Fluorouracil, Epirubicin, Cyclophosphamide, and Trastuzumab	Pre-NAC, 24 weeks post initiation NAC, pre-surgery and 2 weeks post resection	qRT-PCR	Selected from historical laboratory findings
Müller et al^33^	2014	Germany	Serum	127	Prospective (II); Geparquinto Trial (NCT: 00567554)	NAC with Trastuzumab or Lapatinib	Pre-NAC and post-NAC prior to surgery	qRT-PCR	Selected from literature review
Anfossi et al^36^	2014	US	Serum	65	Retrospective (III)	Anti-HER2 therapy	At initiation of treatment	qRT-PCR	Selected from literature review
Liu et al^39^	2019	China	Serum	83	Retrospective (III)	Docetaxel, Paraplatin, and Trastuzumab	Pre-NAC, after second cycle, after completion of NAC, prior to surgery, and during postoperative follow-up	qRT-PCR	Selected from literature review
Wu et al^42^	2012	US	Blood	42	Retrospective (III)	Docetaxel, Doxorubicin, and Cyclophosphamide, vs Doxorubicin and cyclophosphamide, followed by Nab-paclitaxel and Carboplatin	N/R	qRT-PCR	Microarray
Zhu et al^47^	2018	China	Blood	24	Prospective (II); (NCT:02041338)	Epirubicin and Docetaxel	Before initiation of neoadjuvant therapies, after C2 of neoadjuvant treatment and at the time of resection	qRT-PCR	TaqMan© miRNA array
Zhang et al^46^	2020	China	Blood	65	Prospective (II); SHPD001 (NCT:02199418) & SHPH02 (NCT: 02221999)	Paclitaxel, Cisplatin, and Trastuzumab	Before initiation of neoadjuvant therapies	qRT-PCR	Selected from laboratory findings
Sun et al^35^	2017	China	Tumour tissue	39	Retrospective (III)	Docetaxel and Cyclophosphamide	N/R	qRT-PCR	Agilent microarray platform
De Mattos-Arruda et al^41^	2015	Spain	Tumour tissue	52	Retrospective (III)	Anthracycline, Docetaxel, and Trastuzumab	N/R	qRT-PCR	Selected from literature review
Ohzawa et al^44^	2017	Japan	Tumour tissue	47	Retrospective (III)	Anthracycline and Docetaxel	Core biopsy during preoperative treatment	qRT-PCR	Agilent microarray platform
Cataldo et al^45^	2018	Italy	Tumour tissue	52	Retrospective (III)	Trastuzumab	Core biopsy prior to neoadjuvant treatment	qRT-PCR	Selected from previous literature

Abbreviations: C2, second cycle of neoadjuvant treatment; ELISA, enzyme-linked immunosorbent assay; HER2, Human epidermal growth factor receptor-2; miRNA, microRNA; N, number; LOE, level of evidence; NAC, neoadjuvant 1; N/R, not reported; pCR, pathological complete response; qRT-PCR, quantitative real-time polymerase chain reaction; US, United States.

**Table 2.** Circulating micro-RNA expression and their ability to predict response to neoadjuvant therapies.

Author	Year	MicroRNA	Role
Müller et al^33^	2014	miR-21, miR-210, and miR-373	Increased in patient’s serum post-NAC and expression predictive of OS
Di Cosimo et al^38^	2019	miR-140a-5p, miR-148a-3p, and 374a-5p	Increased circulating plasma levels associated with pCR and miR-140a-5p predicted enhanced EFS
Anfossi et al^36^	2014	miR-19a	Increased expression in serum indicating enhanced clinical outcomes in those treated with Trastuzumab
Zhao et al^37^	2011	miR-221	Increased expression in plasma predictive of poor sensitivity Trastuzumab
Di Cosimo et al^34^	2020	miR-376-3p miR-15a-5p, miR-140-3p, miR-320a, miR-320b, miR-363-3p, miR-378a-3p, miR-486-5p, and miR-660-5p miR-30d-5p miR-369-3p miR-26a-5p and miR-374b-5p let-7g-5p and miR-191-5p miR-195-5p	Baseline downregulation associated with pCR rates to Lapatinib Increased expression associated with pCR to Lapatinib at 2 weeks Decreased expression associated with pCR to Lapatinib at 2 weeks Baseline downregulation associated with pCR rates to Trastuzumab Increased expression associated with pCR to Trastuzumab at 2 weeks Increased expression associated with pCR to combined Trastuzumab and Lapatinib at 2 weeks Decreased expression associated with pCR to combined Trastuzumab and Lapatinib at 2 weeks
Liu et al^39^	2019	miR-21	Decreased expression in serum associated with clinical response to NAC
Stevic et al^40^	2018	miR-199a	Aberrant expression associated with pCR to NAC
Wu et al^42^	2012	miR-375 miR-122	Upregulation predicts pCR to NAC Downregulation predicts pCR to NAC
Jung et al^43^	2012	miR-210	Lower expression miR-210 predicted pCR while increased expression was associated with residual disease
Zhu et al^47^	2018	miR-34a	Reduced miR-34a expression was observed in non-responders
Zhang et al^46^	2020	miR-222-3p	Low miR-222-3p expression was predictive of achieving pCR (odds ratio: 0.258, 95% confidence interval: 0.070-0.958, *P* = .043) and favourable DFS and OS

Abbreviations: C2, second cycle of neoadjuvant treatment; DFS, disease-free survival; EFS, event-free survival; HER2, human epidermal growth factor receptor-2; N, number; NAC, neoadjuvant chemotherapy; N/R, not reported; OS, overall survival; pCR, pathological complete response; qRT-PCR, quantitative real-time polymerase chain reaction; US, United States.

**Table 3.** MicroRNA expression and their ability to predict response to neoadjuvant therapies in solid tumour tissue.

Author	Year	MicroRNA	Role
Sun et al^35^	2017	miR-9	Increased therapeutic effect to NAC in HER2 positive disease
De Mattos-Arruda et al^41^	2015	miR-21	Overexpression induced resistance to neoadjuvant combined Trastuzumab and chemotherapy
Ohzawa et al^44^	2017	miR-106b-3p, miR-136-5p, miR-142-5p, miR-150-5p, miR-181a-3p, miR-181c-5p, miR-182-5p, miR-196a-5p, miR-200a-5p, miR-218-5p, miR-301b, miR-342-5p, miR-362-5p, miR-376a-3p, miR-376c-3p, miR-505, miR-582-5p, miR-1180, miR-1238-5p, miR-3609, miR-3620-3p, miR-4418 and miR-4737 let-7a-3p, miR-28, miR-31-3p, miR-34b-5p, miR-148b-3p, miR-151a-3p, miR-152, miR-203, miR-210, miR-376c-3p, miR-377-3p, miR-429, miR-449b-5p, miR-487b, miR-499a, miR-550-5p, miR-1237-3p, miR-3907, miR-4291, miR-5684, miR-6515-3p	Downregulated in those achieving pCR following NAC and Trastuzumab Increased expression in those achieving pCR following NAC and Trastuzumab
Cataldo et al^45^	2018	miR-205	Increased expression in responders to Trastuzamab (61%) vs non-responders (26%)

Abbreviations: HER2, human epidermal growth factor receptor-2; N, number; NAC, neoadjuvant chemotherapy; N/R, not reported; pCR, pathological complete response; qRT-PCR, quantitative real-time polymerase chain reaction; UK, United Kingdom.

### Levels of evidence of included studies

Of included studies, 7 were prospective analyses.^33,34,38,40,43,46,47^ These included participants from 4 studies which encompassed data from 3 phase III randomised clinical trials (NeoALLTO, Geparquinto, and Geparsixto trials),^33,34,38,40^ 2 studies including patients from 3 phase II clinical trials,^46,47^ and 1 prospective study.^43^ The remaining 8 studies included data from retrospective studies.^35-37,39,41,42,44,45^

### MicroRNA expression and response to neoadjuvant therapy

Overall, there were clinical samples from 1335 patients used in this study. Of these, 1145 provided liquid biopsy tissue (ie, blood, serum or plasma) for the evaluation of ct-miRNA (85.8%) and 190 provided solid tumour tissue samples for evaluation (14.2%). Seven studies used a microarray technique to select suitable miRNA for analysis.^34,35,38,40,42,44,47^

The expression levels of 73 different miRNAs correlated with response to neoadjuvant therapy (Tables 2 and 3). Increased expression of 41 miRNA was identified in responders, while expression of 29 miRNA was reduced in responders. Conflicting data from the literature regarding the expression of miR-21, miR-210, and miR-376c-3p were described, with studies indicating both increased and reduced expression of the biomarker to impact patient response to neoadjuvant treatment. Stevic et al^40^ reported ‘aberrant’ expression of miR-199a to be predictive of pCR to NAC in patient plasma. In relation to the expression of miRNA in predicting response to neoadjuvant therapy for different molecular subtypes, the study by Ohzawa et al^44^ was the only study reporting miRNA expression profiles in relation to luminal B – HER2 overexpressing and HER2+ (Table 4). The previously outlined roles of these miRNA in the context of carcinoma are outlined in Table 5.

**Table 4.** MicroRNA expression and their ability to predict response to neoadjuvant therapies based on breast cancer molecular subtypes.

Author	Year	Molecular subtype	Role
Ohzawa et al^44^	2017	Luminal B – HER2 overexpressing	There was increased expression of miR-28, miR-34b-5p, miR-148b-3p, miR-151a-3p, miR-152, miR-203, miR-210, miR-376c-3p, miR-377-3p, miR-429, miR-487b, miR-3907, miR-4291, and miR-5684 in patients achieving a pCR. There was decreased expression of miR-301b, miR-582-5p, and miR-4737 in patients achieving a pCR.
		HER2 overexpressing	There was increased expression of let-7a-3p, miR-550-5p, miR-1237-3p, and miR-6515-3p in patients achieving a pCR. There was decreased expression of miR-106b-3p, miR-136-5p, miR-181a-3p, miR-196a-5p, miR-218-5p, miR-342-5p, miR-362-5p, miR-376a-3p, miR-376c-3p, and miR-505 in patients achieving a pCR.

HER2, human epidermal growth factor receptor-2; pCR, pathological complete response.

**Table 5.** The other functional roles of the mi(cro)RNAs in this systematic review which correlated with response to neoadjuvant therapies.

MicroRNA	Role	Author
Let-7g	Oncomir – invasion and metastasis	Qian et al^48^
miR-9	Tumour suppressor miRNA	Selcuklu et al^49^
miR-21	Oncomir	Yan et al^50^
miR-26a-5p	Tumour suppressor miRNA	Huang et al^51^
miR-122	Oncomir – promotes metastasis	Fong et al^52^
miR-140-3p	Tumour suppressor miRNA	Zhou et al^53^
miR-148a-3p	Regulates angiogenesis	Kim et al^54^
miR-195-5p	Oncomir	Yang et al^55^
miR-199a	Tumour suppressor miRNA	Chen et al^56^
miR-210	Oncomir and breast tumour hypoxia-regulation	Qin et al^57^
miR-221	Oncomir and tumour suppressor miRNA	Garofalo et al^58^
miR-222-3p	Oncomir – proliferation and invasion	Liu et al^59^
miR-320	Tumour suppressor miRNA	Luo et al^60^
miR-320a	Oncomir	Yang et al^61^
miR-373	Oncomir and tumour suppressor miRNA	Wei et al^62^
miR-374a-5p	Oncomir	Son et al^63^
miR-375	Tumour suppressor miRNA	Fu et al^64^
miR-376-3p	Oncomir	An et al^65^
miR-486	Oncomir	Gharehdaghchi et al^66^
miR-660-5p	Tumour suppressor miRNA	Shen et al^67^

### MicroRNA expression from liquid biopsy and tumour tissue

Eleven studies correlated ct-miRNA expression with response to neoadjuvant treatment (Table 2). Of these, 5 evaluated ct-miRNAs expressed in plasma, and 3 measured miRNA in whole blood and serum respectively. Overall, 20 ct-miRNA exhibited increased expression and 6 had decreased expression in responders. Both miR-21 and miR-210 were reported to have increased and decreased expression in the circulation of responders, while miR-199a was expressed inconsistently in the circulation of responders (Table 2). Four studies correlated miRNA expression in tumour tissue in response to neoadjuvant therapies; 21 miRNA demonstrated increased expression while 23 miRNA showed increased expression in the tissue of responders. In addition, miR-376c-3p was reported to have increased and decreased expression in the tumours of responders (Table 3).

### Timepoints of tissue extraction

**Figure 2.**
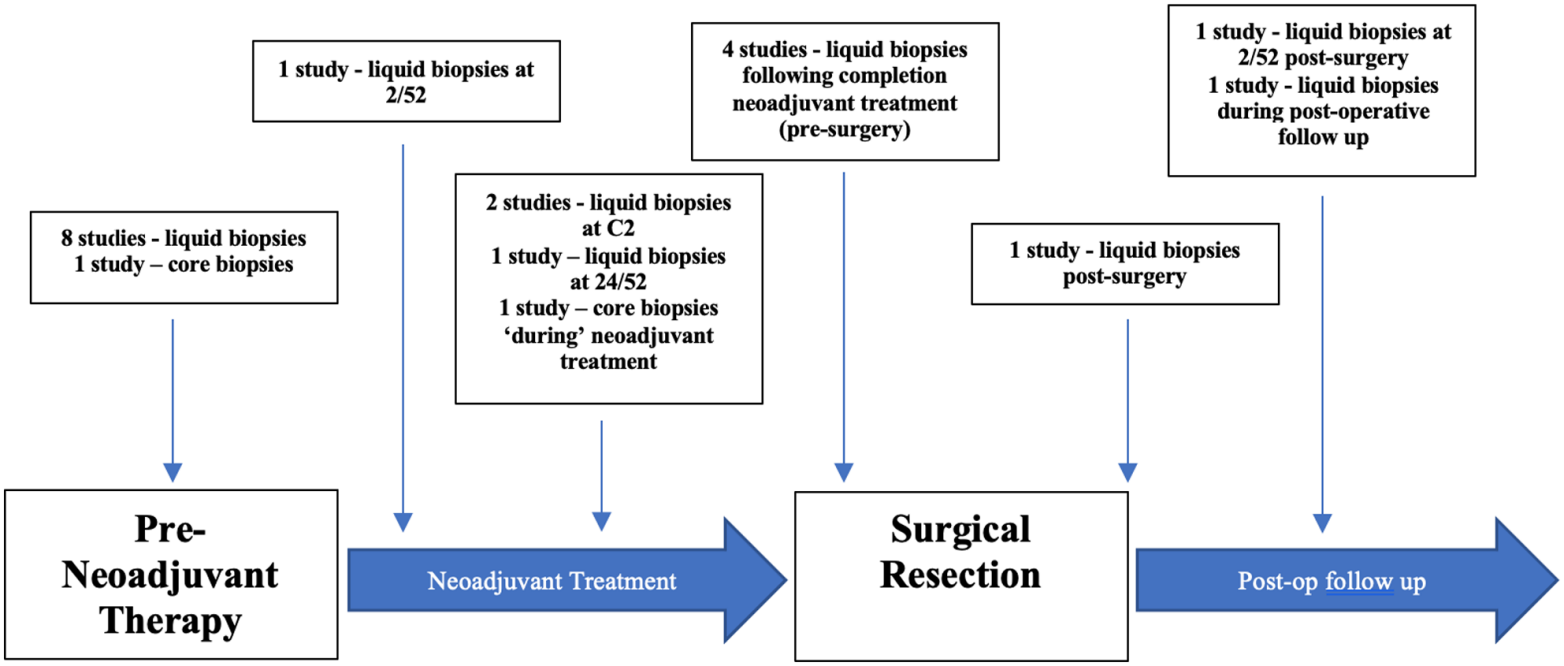
Time points at which liquid and solid tumour biopsies were obtained from patients undergoing neoadjuvant therapies for human epidermal growth factor receptor-2 positive breast cancer in included studies.

Of the 15 included studies, 7 measured miRNA expression at varying timepoints (ie, pre-, during, and post-neoadjuvant therapy)^33,34,38-40,43,47^ (Figure 2). Five studies obtained patient tissue at one timepoint during their study,^36,37,44-46^ while 3 studies did not report the timing of when their samples were taken.^35,41,42^

### Clinicopathological data

The mean age at diagnosis of the 1335 patients included in this study was 55.8 years (range, 21-83 years) with 50.8% of patients being aged 50 years or older at diagnosis (262/516 – 6 studies). Of reported data, 70.4% of tumours were tumour stage 1-2 (280/398 – 4 studies), 56.2% had nodal involvement (338/602), 29.1% were grade 3 (103/354 – 5 studies), and 89.8% had ductal histology (194/216 – 3 studies). Overall, 55.6% were oestrogen receptor positive (luminal B – HER2+) (244/439 – 6 studies) and 62.7% were progesterone receptor positive (138/220 – 3 studies).

## Discussion

This systematic review is the first study which comprehensively highlights the utility of miRNAs as predictive biomarkers in early-stage HER2+ breast cancer. There is emerging evidence indicating that a panel of miRNA may be useful in substratifying patients who are more likely to achieve a response to neoadjuvant therapy,^38,68^ and the current analysis identifies 73 miRNA which may be useful in predicting response to conventional neoadjuvant treatment strategies, including 41 miRNA predictive of an increased likelihood to achieve pCR. pCR is at the epicentre of recent clinical and translation research oncology trials, thus becoming incorporated into the paradigm as the primary analytical endpoint in several multicentre trials of prospective, randomised controlled design.^69-71^ The identification of biomarkers predictive of pCR would serve purpose in aiding preoperative surgical planning, guiding therapeutic strategies, and providing useful prognostic data indicative of oncological and survival outcomes. Therefore, it remains imperative that translational research efforts focus on deciphering those likely to achieve a pCR or to enhance response rates within those diagnosed with early-stage HER2/neu enriched breast carcinoma.

The clinical utility of miRNA signatures may prove useful in patient prognostication, predicting response to the therapies, and augmenting current therapeutic strategies.^29,68^ This is the first systematic review which combines the seminal work of previous authors to select and identify miRNAs with expression profiles capable of predicting response or pCR to neoadjuvant therapies: in the translation research arm of the NeoALTTO trial, measurement of ct-miR-140a-5p, ct-miR-148a-3p, and ct-374a-5p predicted pCR to Trastuzumab (combined predictive ability of 54% vs 0% in patients with increased expression vs reduced expression). In addition, decreased expression of miR-369-3p at baseline enhanced the likelihood of pCR to Trastuzumab, while increased expression of miR-26a-5p and miR-374-5p 2 weeks into treatment was indicative of pCR.^34,38^ In addition, Zhang et al^46^ reported that reduced miR-222-3p expression correlated with pCR. If combined with results of the GeparSixto trial,^40^ molecular profiling of ct-miR-199a within the miRNA signature described by Di Cosimo et al may enhance predictability of response to treatment. Thus, review of these previous studies may prove informative for research groups intending to engage in future translational research studies evaluating the role of miRNA signatures in predicting response to neoadjuvant therapies.

This systematic review also highlights the disagreement between studies as to which miRNA are relevant, and indeed, some studies are in direct conflict as to whether a particular miRNA is oncogenic or tumour suppressive.^33,39,41,43^ This speaks to the ubiquitous and multifunctional nature of miRNA; and the authors must emphasise the multifunctional roles played by these biomarkers in regulating biological processes, as outlined in Table 5. Consequently, it seems imperative that designing a multi-miRNA panel is perhaps the most fruitful and informative means of providing a breakthrough in predicting pCR following neoadjuvant therapies; Di Cosimo et al^38^ identified a 5 miRNA signature with strong and independent sensitivity for response to both Trastuzumab (area under the curve [AUC]: 0.81 [0.70-0.92]) and Lapatinib (AUC: 0.71 [0.55-0.86]) respectively, within HER2+ breast neoplasms in the translational research arm of the NeoALLTO study. Furthermore, within the context of all breast cancers, the seminal work of McGuire et al^68^ also illustrated the predictive value of multiple miRNA in predicting response to NAC. As previously outlined, these studies suggest that novel biomarker expression panels consisting of carefully selected, clinically relevant miRNA may be useful in predicting pCR in setting of breast carcinoma.

However, this review of current evidence highlights the lack of consensus with respect to performing a translational research trial measuring circulatory biomarker levels at different timepoints during neoadjuvant treatment phase. In the GeparSixto trial,^40^ Stevic et al describe results based on ct-miRNA expression on pre- and post-NAC plasma, which provides informative results in relation to response to treatment. Anfossi et al^36^ performed tissue sampling at the initiation of therapy administration and failed to perform further liquid or tissue biopsies during their study, while Jung performed interval serum sampling during the neoadjuvant, surgical, and adjuvant phases of treatment in their study.^43^ Interestingly in NeoALTTO,^34^ second liquid biopsy was performed just 2 weeks into neoadjuvant therapy, providing informative data in relation to oncological outcome and response to treatment. The next generation of prospective multicentre, translational research collaborations can learn from the work of Di Cosimo et al, given their insight into the value of ct-miRNA as pertinent biomarkers in predicting response of breast cancers to neoadjuvant therapy, when measured at early phases of administrating neoadjuvant therapies.

The current systematic review outlines the methodological details of previous studies addressing the role of miRNA to predict response to neoadjuvant therapies in HER2+ breast cancer. One of the inherent challenges with miRNA measurement is the current ambiguity and uncertainty surrounding the most appropriate circulating medium from which to measure them, which is evident from the fact that 5 used plasma, while 3 measured miRNA in whole blood and serum respectively. The use of real-time quantitative reverse transcription polymerase chain reaction (qRT-PCR) is a validated means of miRNA expression appraisal based on the details from previous studies, with all but one study using qRT-PCR. While qRT-PCR is verified, it is believed to be only of relevance in academic and research settings – the absolute quantification of miRNA and validation of their measurement across laboratories remain a future aspiration.^72^ Moreover, 4 of the 7 prospective analyses (including GeparSixto and NeoALLTO trials) used microarray techniques to identify non-coding miRNA targets. The utilisation of miRNA microarray has several benefits versus formal literature review: Microarray records measurements of the relative concentrations of miRNA expression profile within a pre-selected tissue of interest^73,74^ (ie, HER2+ breast tumour tissue, previously acquired from core biopsy), which subsequently facilitates an increased likelihood of identifying molecularly appropriate targets, which increases the possibility of yielding clinically pertinent results. Therefore, the use of microarray in tandem with previous formal literature reviews offers the scientist the ability to construe the most informative data when setting the foundations for future miRNA-based translational research studies.

This systematic review is subject to the inherent limitation of encompassing studies of varying methodology, laboratory techniques, and tissue of such variation. Furthermore, variance in statistical tests and analyses performed within studies limit decisive conclusions. The most appropriate timing for the acquisition of liquid or tumour biopsies is yet to be elucidated, with included studies providing conflicting data pertaining to the most appropriate tissue to be extracted. Furthermore, consensus in relation to whether liquid or core biopsies are more informative remains. Finally, varying neoadjuvant therapeutic regimens and strategies were deployed in the included studies, limiting the congruence of results suggesting cautious interpretation of these results is required. In spite of these limitations, the authors acknowledge this systematic review elucidates all previous translational research studies and their miRNA predictive of sensitivity to therapy in HER2+ tumours.

Despite considerable funding, investment, and resource distribution into the modern translational research effort, we are yet to discover novel biomarkers that can rival the principal ER, PgR, and HER2/neu receptors in providing prognostication and directing therapeutic decision making. Research efforts would still continue to assess in vivo predictive biomarkers of pCR to minimise host toxicity, while inducing tumour-specific cytotoxicity and de-escalation of therapy in non-responder patients. This systematic review is the first to provide an extensive overview of miRNA validated in previous in vivo studies with the potential of predicting favourable response to current neoadjuvant treatment strategies for patients being treated for HER2+ breast cancer. In addition, this study accentuates successful facets of previous studies evaluating the role of miRNA in the neoadjuvant setting, while highlighting several points of contention to be addressed in future prospective translational studies in the space. Thus, the requirement for an informative consensus illustrating the role and overarching potential of miRNA in predicting response to neoadjuvant therapies in HER2+ breast cancer remains. Perhaps the next generation of prospective, translational research studies will incorporate both venous and core biopsy sampling at time intervals as an endeavour to discover prospective and stable circulating biomarkers, such as miRNAs, which may indicate pCR to therapies within HER2/neu overexpressing breast cancer.
